# Mitochondrial bioenergetics are not associated with myofibrillar protein synthesis rates

**DOI:** 10.1002/jcsm.13532

**Published:** 2024-07-15

**Authors:** Andrew M. Holwerda, Marlou L. Dirks, Pierre‐Andre Barbeau, Joy Goessens, Annemie Gijsen, Luc J.C. van Loon, Graham P. Holloway

**Affiliations:** ^1^ NUTRIM School of Nutrition and Translational Research in Metabolism Maastricht University Medical Centre+ Maastricht The Netherlands; ^2^ Department of Human Health and Nutritional Sciences University of Guelph Guelph Canada; ^3^ Department of Public Health and Sport Sciences, Faculty of Health and Life Sciences University of Exeter Exeter UK

**Keywords:** Aging, Muscle protein synthesis, Physical inactivity, Reactive oxygen species, Skeletal muscle

## Abstract

**Background:**

Mitochondria represent key organelles influencing cellular homeostasis and have been implicated in the signalling events regulating protein synthesis.

**Methods:**

We examined whether mitochondrial bioenergetics (oxidative phosphorylation and reactive oxygen species (H_2_O_2_) emission, ROS) measured *in vitro* in permeabilized muscle fibres represent regulatory factors for integrated daily muscle protein synthesis rates and skeletal muscle mass changes across the spectrum of physical activity, including free‐living and bed‐rest conditions: *n* = 19 healthy, young men (26 ± 4 years, 23.4 ± 3.3 kg/m^2^) and following 12 weeks of resistance‐type exercise training: *n* = 10 healthy older men (70 ± 3 years, 25.2 ± 2.1 kg/m^2^). Additionally, we evaluated the direct relationship between attenuated mitochondrial ROS emission and integrated daily myofibrillar and sarcoplasmic protein synthesis rates in genetically modified mice (mitochondrial‐targeted catalase, MCAT).

**Results:**

Neither oxidative phosphorylation nor H_2_O_2_ emission were associated with muscle protein synthesis rates in healthy young men under free‐living conditions or following 1 week of bed rest (both *P* > 0.05). Greater increases in GSSG concentration were associated with greater skeletal muscle mass loss following bed rest (*r* = −0.49, *P* < 0.05). In older men, only submaximal mitochondrial oxidative phosphorylation (corrected for mitochondrial content) was positively associated with myofibrillar protein synthesis rates during exercise training (*r* = 0.72, *P* < 0.05). However, changes in oxidative phosphorylation and H_2_O_2_ emission were not associated with changes in skeletal muscle mass following training (both *P* > 0.05). Additionally, MCAT mice displayed no differences in myofibrillar (2.62 ± 0.22 vs. 2.75 ± 0.15%/day) and sarcoplasmic (3.68 ± 0.35 vs. 3.54 ± 0.35%/day) protein synthesis rates when compared with wild‐type mice (both *P* > 0.05).

**Conclusions:**

Mitochondrial oxidative phosphorylation and reactive oxygen emission do not seem to represent key factors regulating muscle protein synthesis or muscle mass regulation across the spectrum of physical activity.

## Introduction

Skeletal muscle tissue represents approximately 40% of total body mass and is essential for locomotion, overall metabolism, and glucose homeostasis. Skeletal muscle mass is regulated by the balance between muscle protein synthesis and breakdown. One of the most significant changes with aging is the loss of skeletal muscle mass and strength, termed sarcopenia.^1^ Older individuals display an attenuated muscle protein synthetic response to anabolic stimuli (i.e., food intake and physical activity). This so‐called anabolic resistance results in a negative muscle protein balance, which, at least partly, explains the progression of sarcopenia.

One of the proposed mechanisms contributing to anabolic resistance and sarcopenia is an alteration in mitochondrial bioenergetics. Mitochondria are small organelles that are required for aerobic ATP production and play a central role in redox biology. It is currently debated to what extent mitochondria have a role in the development of sarcopenia. For instance, some studies have demonstrated that aged skeletal muscle tissue displays a lower mitochondrial content,^2^, ^3^ potentially due to mitochondrial‐mediated apoptosis.^4^ However, the degree of mitochondrial content seems to be largely dictated by physical activity level and not age *per se*, which may explain the discrepancies in the literature.^5^, ^6^, ^7^

Increased mitochondrial reactive oxygen species (ROS) emission, in particular, has been associated with motor unit loss,^8^ muscle fibre atrophy,^9^, ^10^ and lower skeletal muscle mass^2^, ^3^ in various rodent models. Additionally, transgenic models that decrease the capacity of mitochondria to produce ATP dismantle neuromuscular junctions and trigger distal degeneration of motor neurons, which is a hallmark of pathological sarcopenia.^11^ While cause‐and‐effect relationships are difficult to establish in humans, the skeletal muscle of older individuals displays lower oxidative phosphorylation and higher mitochondrial ROS emission in the presence of nonsaturating ADP concentrations.^12^ These data support the suggestion that alterations in mitochondria contribute to the development of sarcopenia.

A central factor responsible for age‐related impairments in skeletal muscle mass regulation and metabolic function is the decline in physical activity observed over the lifespan,^13^ which may exacerbate anabolic resistance and accelerate the development of sarcopenia in part as a result of decreased mitochondrial content.^14^ In particular, short‐term periods of inactivity decrease markers of mitochondrial content and oxidative phosphorylation while increasing ROS emission in human and rodent skeletal muscle.^10^, ^15^, ^16^ While several lines of evidence suggest an association between mitochondrial content and metabolic health, the relationship between the intrinsic mitochondrial function and protein synthesis in humans remains to be fully established. However, in the absence of changing mitochondrial content, pharmacological and genetic approaches that decrease the intrinsic capacity for mitochondrial ROS emission attenuate muscle atrophy during inactivity,^10^, ^17^, ^18^ disuse atrophy,^19^ and aging^20^ in rodents. While these data may suggest that therapeutic strategies that attenuate mitochondrial ROS are beneficial, these are not uniform findings.^21^ The inconsistency in the literature highlights the importance of interrogating the broader role for mitochondrial bioenergetics in maintaining muscle tissue remodelling to promote healthy aging.

Given the current knowledge gaps, we examined whether impaired intrinsic mitochondrial bioenergetics (i.e., lower oxidative phosphorylation or greater ROS emission) are related to skeletal muscle protein synthesis rates (1) in healthy younger individuals, (2) in response to physical inactivity (bed rest), and (3) following resistance‐type exercise training in older humans. Additionally, as proof‐of‐principle, (4) we investigated the relationship between attenuated mitochondrial ROS emission and myofibrillar and sarcoplasmic protein synthesis rates in genetically modified mice [mitochondrial‐targeted catalase (MCAT)]. In all instances, we hypothesized that lower mitochondrial‐specific oxidative phosphorylation and/or greater mitochondrial specific ROS emission would be associated with lower daily myofibrillar protein synthesis rates and unfavourable changes in skeletal muscle mass.

## Methods

### Ethical approval

Experimental procedures for all human experiments were approved by the Medical Ethical Committee of the Maastricht University Medical Center+, the Netherlands, and conformed to standards for the use of human subjects in research as outlined in the most recent version of the Declaration of Helsinki. We were interested in investigating the relationship between mitochondrial bioenergetics and muscle protein synthesis and thereby utilized two separate datasets with overlapping analysis. Specifically, we identified a total of 19 (*n* = 19) healthy, young men (26 ± 4 years, 23.4 ± 3.3 kg/m^2^) and 10 (*n* = 10) healthy older men (70 ± 3 years, 25.2 ± 2.1 kg/m^2^) with mitochondrial bioenergetics and skeletal muscle myofibrillar fractional synthetic rates measurements already performed, with sufficient remaining frozen muscle to determine mitochondrial DNA (mtDNA). As such, the data for the younger^15^, ^22^ and older men^12^, ^23^ were previously published. Subject characteristics of the test participants are presented in Table 1.

**Table 1:** Subjects' characteristics

	Young (*n* = 19)	Old (*n* = 10)	*P*‐value
Age, year	26 ± 4	70 ± 3	<0.001
Body mass, kg	75.4 ± 12.6	76.8 ± 8.4	0.691
Height, m	1.79 ± 0.08	1.75 ± 0.06	0.081
BMI, kg/m^2^	23.4 ± 3.3	25.2 ± 2.1	0.090
Body fat, %	22.1 ± 4.5	20.4 ± 2.3	0.338
Lean body mass, kg	55.9 ± 8.8	59.6 ± 5.1	0.207
Fasting glucose, mmol/L	5.0 ± 0.7	5.8 ± 0.4	0.001
Fasting insulin, mU/L	9.4 ± 4.7	8.9 ± 4.6	0.932
HOMA‐IR	2.1 ± 1.2	2.3 ± 1.1	0.532
HbA1c, %	5.2 ± 0.4	5.4 ± 0.3	0.007

Values are means ± SDs. *n* = 19 for young and *n* = 10 for old. Data were analysed using a Student's unpaired *t*‐test. Significance was set at *P* < 0.05.

BMI, body mass index; HbA1c, glycated haemoglobin; HOMA‐IR, homeostatic model assessment of insulin resistance.

All subjects were informed of the nature and possible risks of the experimental procedures before their written informed consent was obtained. The bed rest trial in younger men was registered at ClinicalTrials.gov (www.clinicaltrials.gov) as NCT02521025, and the training study in older men was registered on the Netherlands Trial Registry (www.trialregister.nl) as NTR5082.

For the animal study, C57BL/6J wild type (WT, *n* = 8) and mitochondrial catalase transgenic (MCAT, *n* = 8) male mice were purchased from The Jackson Laboratory (Bar Harbor, USA) at 8 weeks of age. Animals were single‐housed on a 12:12‐h light:dark cycle within a temperature‐regulated environment with unrestricted access to a standard chow diet, and water available *ad libitum*, until they reached 15 weeks of age. Experimental procedures used during animal experimentation were approved by the Animal Care Committee at the University of Guelph (protocol number: AUP4241) and followed the guide for the care and use of laboratory animals, as published by the National Institutes of Health.

### Pretesting and study design

All human participants were screened for medical issues and excluded if any gastrointestinal, cardiovascular, neurological, or renal diseases were present. The younger participants were subjected to 1 week of bed rest while being tube‐fed in energy balance (continuous feeding, *n* = 9; bolus feeding, *n* = 10).^22^ The older participants performed supervised progressive resistance‐type exercise training three times per week for a 12‐week period, consisting of three or four sets (8 to 10 repetitions per set) of leg press and leg extension, in addition to upper body exercises (protein supplementation, *n* = 5; placebo supplementation, *n* = 5).^23^

### Anthropometry, body composition, and quadriceps cross sectional area

Anthropometric measurements were assessed using standardized procedures: body mass by a digital scale to within 100 g, and height by a stadiometer to within 0.5 cm. Body composition was measured at the whole‐body and regional level using DXA (Discovery A; Hologic, USA). Anatomic cross‐sectional area (CSA) of the quadriceps muscle was assessed by computed tomography (CT) scanning (Philips Brilliance 64; Philips Medical Systems) before and after the bed rest and training interventions, as described previously.^23^ Briefly, a 3‐mm‐thick axial image was made at 15 cm above the patella, with participants in supine position, while their legs were extended, and their feet were secured. CT scans were analysed for quadriceps muscle CSA by manual tracing using ImageJ software (version 1.50c, National Institutes of Health, Bethesda, MD).^24^

### Muscle biopsy sampling

Muscle biopsy samples were obtained before and after the week preceding bed rest, the week of bed rest (younger participants), and before and after the 12th week of training (older participants) from the middle region of the vastus lateralis, 15 cm above the patella and 4 cm below entry through the fascia, using the percutaneous needle biopsy technique.^25^

### Muscle fibre cross‐sectional area

Muscle biopsy specimens collected from the older individuals were sliced into 5‐mm‐thick cryosections and stained for muscle fibre type, and type I and II muscle fibre CSA and composition were determined as described previously.^23^

### Myofibrillar fractional synthesis rates

For the human studies, deuterium oxide (^2^H_2_O, Cambridge Isotopes Laboratories, Tewksbury, MA) was provided throughout the duration of the week preceding bed rest, the week of bed rest (younger participants), and during week 12 of exercise training (older participants) as previously described.^22^, ^23^

For the rodent study (WT vs. MCAT), mice received one intraperitoneal injection of 99% deuterium oxide at 0.015 mL/g body mass and had access to 4% deuterium‐enriched drinking water for the duration of the assessment period (7 days). The assessment period ended with soleus excision under anaesthetic (2% isoflurane and 98% oxygen).

For both human and rodent studies, myofibrillar protein fractions were isolated from the pre‐ and post‐intervention muscle samples. The samples were derivatized and the increase in ^2^H‐alanine enrichment was assessed using a GC‐pyrolysis‐isotope‐ratio mass spectrometer for the human studies^26^ and a GC–MS for the rodent study.^27^ Myofibrillar protein synthesis rates were calculated as previously described.^26^, ^27^

### Mitochondrial bioenergetics

Saponin‐permeabilized muscle fibres were generated and measurements of mitochondrial oxidative phosphorylation and reactive oxygen species emission (H_2_O_2_) were determined in buffer Z supplemented with 20 mM Amplex Red (Invitrogen, Carlsbad, CA, USA), 5 U/mL horseradish peroxidase, 40 U/mL superoxide dismutase, and 25 μM blebbistatin, using an Oxygraph‐2 K with a fluorometry modular attachment (Oroboros Instruments, Innsbruck, Austria) as previously reported.^12^, ^28^, ^29^, ^30^, ^31^ Experiments were conducted at 37°C in 250 mM O_2_ and H_2_O_2_ emission and oxidative phosphorylation values were determined under the presence of 5 mM Pyruvate, 1 mM Malate, and 20 mM Succinate, and the absence (maximal H_2_O_2_) or presence of ADP (25 μM for submaximal H_2_O_2_ and oxidative phosphorylation, 10 000 μM for maximal oxidative phosphorylation). Following substrate titrations, 10 mM cytochrome c was added to ensure outer mitochondrial membrane integrity. Samples from the human studies were subjected to various concentrations of ADP to determine oxidative phosphorylation and ROS emission under submaximal and maximal muscle tissue conditions.^12^ For the human studies, values from the titration were used to model the *in vivo* muscle tissue environment. For the mouse study, mitochondrial H_2_O_2_ was supported by 20 mM succinate, and determined in the presence and absence of 100 μM ADP as previously reported (Lumina, Thermo Scientific, Waltham, MA, USA).^31^ The rate of H_2_O_2_ emission was calculated from a standard curve established with the same reaction conditions after subtracting the fibre background. All fibres were weighed in buffer Z before the start of an experiment to normalize oxidative phosphorylation to muscle bundle weight. Values from the human experiments were also dual normalized to mitochondrial DNA (mtDNA) to determine the mitochondrial specific/intrinsic bioenergetic function, which was determined by qPCR (mitochondrial gene: NADH dehydrogenase 1, nd1; nuclear gene: lipoprotein lipase, LDL). Mitochondrial DNA values were calculated using the −2^∆∆Ct^ method.^32^

### Glutathione measurements

A small (30–50 mg) portion of each muscle biopsy was homogenized on ice in a 1:10 *w*/*v* ratio with phenanthroline dissolved in 7% PCA to determine GSH and GSSG content on a high‐performance liquid chromatograph (HPLC) (column: Microsorb 100‐5), as previously reported.^33^ Final GSH and GSSG muscle concentrations were determined by correcting for sample dilution that occurred throughout the preparation phases. The sum of GSH and GSSG concentrations represents total glutathione.

### Statistical analysis

All data are expressed as means ± SDs. For the human participants, Student's unpaired two‐tailed *t* tests were applied to determine differences in baseline characteristics between age groups. Pearson's *r* moment correlations were performed to determine whether associations existed between maximal and submaximal mitochondrial oxidative phosphorylation, maximal and submaximal H_2_O_2_ emission rates, total glutathione concentrations and GSH:GSSG and myofibrillar protein synthesis rates and the change in quadriceps CSA over bed rest and training interventions. For the WT and MCAT mice, Student's unpaired two‐tailed *t* tests were applied to determine differences in maximal mitochondrial oxidative phosphorylation, maximal and submaximal H_2_O_2_ emission rates, myofibrillar, and sarcoplasmic protein synthesis rates. Pearson's *r* moment correlations were performed to determine whether associations existed between maximal mitochondrial oxidative phosphorylation, maximal, and submaximal H_2_O_2_ emission rates and daily myofibrillar and sarcoplasmic fractional synthetic rates. Significance was set at *P* < 0.05. Calculations were performed with the use of SPSS (version 21.0; IBM Corporation).

## Results

### Baseline mitochondrial bioenergetics and muscle protein synthesis rates in young men

**Figure 1:**
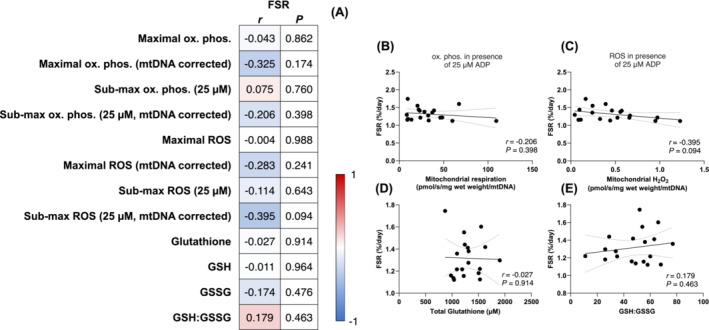
Associations between daily myofibrillar protein synthesis rates (FSR, %/day) and maximal and submaximal oxidative phosphorylation and (pmol O_2_/s/mg wet weight), mitochondrial specific maximal and submaximal oxidative phosphorylation (pmol O_2_/s/mg wet weight/mtDNA), maximal and submaximal H_2_O_2_ emission (pmol H_2_O_2_/s/mg wet weight), mitochondrial specific maximal and submaximal H_2_O_2_ emission (pmol H_2_O_2_/s/mg wet weight/mtDNA), total glutathione (uM), GSH (uM), GSSG (uM), and GSH:GSSG in healthy young men (*n* = 19) at baseline (A). H_2_O_2_ emission and oxidative phosphorylation values were determined under the presence of 5 mM pyruvate, 1 mM malate, and 20 mM succinate, and the absence (maximal H_2_O_2_) or presence of ADP (25 μM for submaximal H_2_O_2_ and oxidative phosphorylation, 10 000 μM for maximal oxidative phosphorylation). Data were analysed using Pearson's *r* product moment correlations. Values in panel (A) represent the *P* values and correlation coefficients (*r* values), which are colour‐coded to represent negative (blue) and positive (red) correlations. *Significant correlations (*P* < 0.05) are in bold text. Selected correlations are also displayed between mitochondrial specific submaximal oxidative phosphorylation (B, pmol O_2_/s/mg wet weight/mtDNA), mitochondrial specific submaximal H_2_O_2_ emission (C, pmol H_2_O_2_/s/mg wet weight/mtDNA), total glutathione (D, μM), GSH:GSSG (E) and daily myofibrillar protein synthesis rates (%/day). The solid lines represent the linear regression lines of best fit and the dashed lines represent the 95% confidence intervals.

We first examined whether mitochondrial specific bioenergetics are associated with daily myofibrillar fractional synthetic rates in healthy younger men at baseline. We did not observe any associations between myofibrillar protein synthesis rates and assessments of mass‐specific mitochondrial bioenergetics, intrinsic mitochondrial bioenergetics, or indices of cellular redox stress (Figure 1). In particular, myofibrillar protein synthesis rates did not correlate with intrinsic mitochondrial submaximal oxidative phosphorylation (Figure 1B), intrinsic mitochondrial submaximal H_2_O_2_ emission (Figure 1C), total glutathione (Figure 1D) or GSH:GSSG (Figure 1E) (all *P* > 0.05). Altogether, these data illustrate that intrinsic mitochondrial bioenergetics are not associated with muscle protein synthesis rates in healthy young individuals at baseline.

### Mitochondrial bioenergetics and muscle protein synthesis rates following bed rest

**Figure 2:**
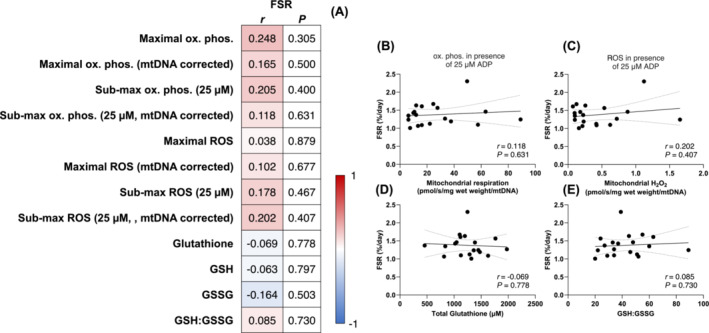
Associations between daily myofibrillar protein synthesis rates (FSR, %/day) and maximal and submaximal oxidative phosphorylation and (pmol O_2_/s/mg wet weight), mitochondrial specific maximal and submaximal oxidative phosphorylation (pmol O_2_/s/mg wet weight/mtDNA), maximal and submaximal H_2_O_2_ emission (pmol H_2_O_2_/s/mg wet weight), mitochondrial specific maximal and submaximal H_2_O_2_ emission (pmol H_2_O_2_/s/mg wet weight/mtDNA), total glutathione (μM), GSH (μM), GSSG (μM), and GSH:GSSG in healthy young men (*n* = 19) following 1 week of strict bed rest (A). H_2_O_2_ emission and oxidative phosphorylation values were determined under the presence of 5 mM pyruvate, 1 mM malate, and 20 mM succinate, and the absence (maximal H_2_O_2_) or presence of ADP (25 μM for submaximal H_2_O_2_ and oxidative phosphorylation, 10,000 μM for maximal oxidative phosphorylation). Data were analysed using Pearson's *r* product moment correlations. Values displayed in panel (A) represent the *P* values and correlation coefficients (*r* values), which are colour‐coded to represent negative (blue) and positive (red) correlations. *Significant correlations (*P* < 0.05) are in bold text. Selected correlations are also displayed between mitochondrial specific submaximal oxidative phosphorylation (B, pmol O_2_/s/mg wet weight/mtDNA), mitochondrial specific submaximal H_2_O_2_ emission (C, pmol H_2_O_2_/s/mg wet weight/mtDNA), total glutathione (D, μM), GSH:GSSG (E) and daily myofibrillar protein synthesis rates (%/day). The solid lines represent the linear regression lines of best fit and the dashed lines represent the 95% confidence intervals.

Given the concurrent dysregulation of mitochondrial bioenergetics and muscle protein synthesis rates following short‐term disuse, we next examined potential associations in younger adults following 1 week of bed rest. Similar to our findings at baseline, mitochondrial bioenergetics (mass and intrinsic specific) and markers of redox stress did not correlate with myofibrillar protein synthesis rates following bedrest (Figure 2). Specifically, intrinsic mitochondrial submaximal oxidative phosphorylation (Figure 2B), intrinsic mitochondrial submaximal H_2_O_2_ emission (Figure 2C), total glutathione (Figure 2D) and GSH:GSSG (Figure 2E) were not associated with daily myofibrillar protein synthesis rates following bed rest (all *P* > 0.05). Altogether, these data illustrate that mitochondrial bioenergetics are not associated with muscle protein synthesis rates following short‐term physical inactivity in healthy young individuals.

**Figure 3:**
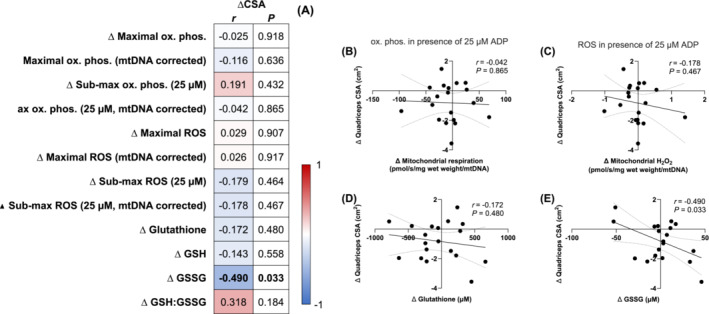
Associations between changes in quadriceps cross‐sectional area (cm^2^) and changes maximal and submaximal oxidative phosphorylation and (pmol O_2_/s/mg wet weight), mitochondrial specific maximal and submaximal oxidative phosphorylation (pmol O_2_/s/mg wet weight/mtDNA), maximal and submaximal H_2_O_2_ emission (pmol H_2_O_2_/s/mg wet weight), mitochondrial specific maximal and submaximal H_2_O_2_ emission (pmol H_2_O_2_/s/mg wet weight/mtDNA), total glutathione (μM), GSH (μM), GSSG (μM), and GSH:GSSG in young men (*n* = 19) following 1 week of strict bed rest (A). H_2_O_2_ emission and oxidative phosphorylation values were determined under the presence of 5 mM pyruvate, 1 mM malate, and 20 mM succinate, and the absence (maximal H_2_O_2_) or presence of ADP (25 μM for submaximal H_2_O_2_ and oxidative phosphorylation, 10,000 μM for maximal oxidative phosphorylation). Data were analysed using Pearson's *r* product moment correlations. Values displayed in panel (A) represent the *P* values and correlation co‐efficients (*r* values), which are colour‐coded to represent negative (blue) and positive (red) correlations. *Significant correlations (*P* < 0.05) are in bold text. Selected correlations are also displayed between changes in mitochondrial specific submaximal oxidative phosphorylation (B, pmol O_2_/s/mg wet weight/mtDNA), mitochondrial specific submaximal H_2_O_2_ emission (C, pmol H_2_O_2_/s/mg wet weight/mtDNA), total glutathione (D, μM), GSH:GSSG (E) and changes in quadriceps cross‐sectional area (cm^2^). The solid lines represent the linear regression lines of best fit and the dashed lines represent the 95% confidence intervals. CSA, cross‐sectional area.

To further interrogate the possible involvement of mitochondrial bioenergetics in regulating muscle mass during short‐term disuse, we examined the potential for changes in mitochondrial bioenergetics to correlate with changes in skeletal muscle mass (quadriceps CSA) following 1 week of bed rest in young men. However, similar to the lack of relationships with myofibrillar protein synthesis rates (Figures 1 and 2), changes in mitochondrial bioenergetics were not related with changes in quadriceps CSA (Figure 3A–C). In line, changes in total glutathione were also not related to changes in quadriceps CSA (*P* > 0.05, Figure 3D). In contrast, changes in GSSG, a marker of redox stress, were negatively associated with changes in quadriceps CSA following bed rest (*r* = −0.490, *P* = 0.033; Figure 3E). These data indicate that while mitochondrial bioenergetics may not be directly associated with muscle protein synthesis rates, disturbances in cellular redox status may have a broader impact on regulating changes in skeletal muscle mass during short‐term disuse.

### Mitochondrial bioenergetics and muscle protein synthesis rates in older men

**Figure 4:**
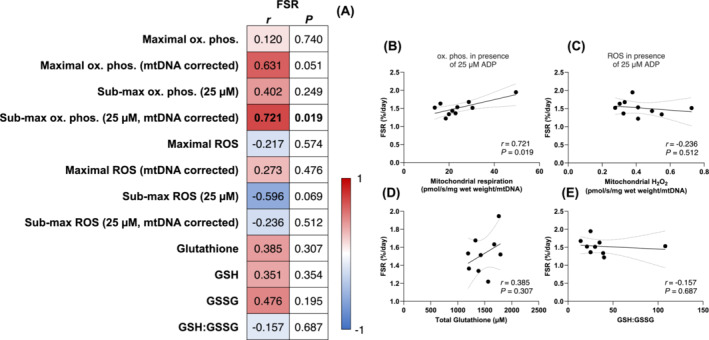
Associations between daily myofibrillar protein synthesis rates (FSR, %/day) and maximal and submaximal oxidative phosphorylation and (pmol O_2_/s/mg wet weight), mitochondrial specific maximal and submaximal oxidative phosphorylation (pmol O_2_/s/mg wet weight/mtDNA), maximal and submaximal H_2_O_2_ emission (pmol H_2_O_2_/s/mg wet weight), mitochondrial specific maximal and submaximal H_2_O_2_ emission (pmol H_2_O_2_/s/mg wet weight/mtDNA), total glutathione (μM), GSH (μM), GSSG (μM), and GSH:GSSG in older men (*n* = 10) following resistance exercise training (A). H_2_O_2_ emission and oxidative phosphorylation values were determined under the presence of 5 mM pyruvate, 1 mM malate, and 20 mM succinate, and the absence (maximal H_2_O_2_) or presence of ADP (25 μM for submaximal H_2_O_2_ and oxidative phosphorylation, 10,000 μM for maximal oxidative phosphorylation). Data were analysed using Pearson's *r* product moment correlations values displayed in panel (A) represent the *P* values and correlation coefficients (*r* values), which are colour‐coded to represent negative (blue) and positive (red) correlations. *Significant correlations (*P* < 0.05) are in bold text. Selected correlations are also displayed between mitochondrial specific submaximal oxidative phosphorylation (B, pmol O_2_/s/mg wet weight/mtDNA), mitochondrial specific submaximal H_2_O_2_ emission (C, pmol H_2_O_2_/s/mg wet weight/mtDNA), total glutathione (D, μM), GSH:GSSG (E) and daily myofibrillar protein synthesis rates (%/day). The solid lines represent the linear regression lines of best fit and the dashed lines represent the 95% confidence intervals.

**Figure 5:**
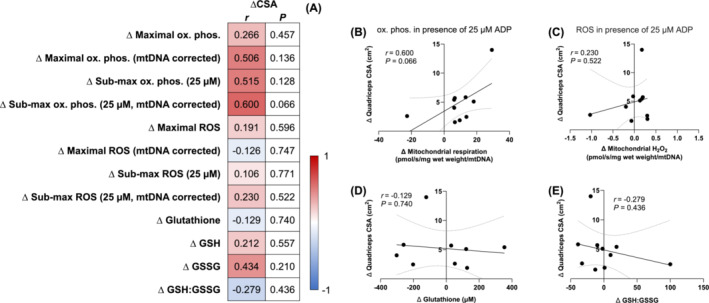
Associations between changes in quadriceps cross‐sectional area (cm^2^) and changes in maximal and submaximal oxidative phosphorylation and (pmol O_2_/s/mg wet weight), mitochondrial specific maximal and submaximal oxidative phosphorylation (pmol O_2_/s/mg wet weight/mtDNA), maximal and submaximal H_2_O_2_ emission (pmol H_2_O_2_/s/mg wet weight), mitochondrial specific maximal and submaximal H_2_O_2_ emission (pmol H_2_O_2_/s/mg wet weight/mtDNA), total glutathione (μM), GSH (μM), GSSG (μM), and GSH:GSSG in older men (*n* = 10) following 12 weeks of progressive resistance exercise training (A). H_2_O_2_ emission and oxidative phosphorylation values were determined under the presence of 5 mM pyruvate, 1 mM malate, and 20 mM succinate, and the absence (maximal H_2_O_2_) or presence of ADP (25 μM for submaximal H_2_O_2_ and oxidative phosphorylation, 10,000 μM for maximal oxidative phosphorylation). Data were analysed using Pearson's *r* product moment correlations. Values displayed in panel (A) represent the *P* values and correlation coefficients (*r* values), which are colour‐coded to represent negative (blue) and positive (red) correlations. *significant correlations (*P* < 0.05) are in bold text. Selected correlations are also displayed between changes in mitochondrial specific submaximal oxidative phosphorylation (B, pmol O_2_/s/mg wet weight/mtDNA), mitochondrial specific submaximal H_2_O_2_ emission (C, pmol H_2_O_2_/s/mg wet weight/mtDNA), total glutathione (D, μM), GSH:GSSG (E) and changes in quadriceps cross‐sectional area (cm^2^). The solid lines represent the linear regression lines of best fit and the dashed lines represent the 95% confidence intervals. CSA, cross‐sectional area.

Next, we investigated the role of mitochondrial bioenergetics in regulating myofibrillar protein synthesis rates with aging. Since a sedentary lifestyle behaviour (and the resulting decline in skeletal muscle mitochondrial content) can confound the interpretation of aging, we examined possible relationships following 12 weeks of progressive resistance training. We observed a positive association between myofibrillar protein synthesis rates and intrinsic mitochondrial submaximal oxidative phosphorylation (*r* = 0.721, *P* = 0.019, Figure 4B). In contrast, we did not observe associations between protein synthesis rates and submaximal intrinsic mitochondrial H_2_O_2_ emission (Figure 4C) and markers of cellular redox stress (*P* > 0.05, Figure 4D,E). While the association between mitochondrial specific submaximal oxidative phosphorylation and myofibrillar protein synthetic rates may imply that mitochondrial respiratory capacity plays a role in facilitating skeletal muscle remodelling during training in older individuals, the lack of associations between the other mitochondrial bioenergetic read‐outs suggests that the relationship is not robust. In support of this, changes in markers of redox stress and mitochondrial bioenergetics, including intrinsic submaximal mitochondrial H_2_O_2_ emission, were not associated with changes in quadriceps cross‐sectional area over the duration of the 12‐week training intervention (Figure 5, all *P* > 0.05). The lack of strong associations was also observed between changes in mitochondrial bioenergetic readouts and changes in mixed, Type I, and Type II muscle fibre CSA, as only submaximal oxidative phosphorylation negatively correlated with the change in Type I fibre CSA (*r* = −0.71, *P* = 0.02; Table S1). Combined, these data indicate that mitochondrial bioenergetics are not associated with muscle mass regulation during exercise training in older individuals.

### Muscle protein synthesis rates in mitochondrial catalase transgenic versus wild‐type mice

**Figure 6:**
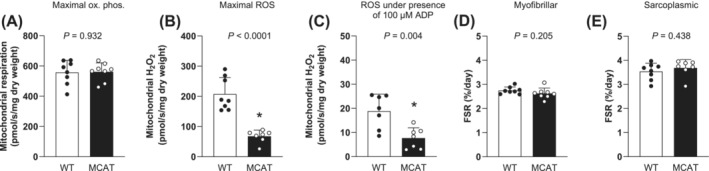
Resting maximal oxidative phosphorylation (A, pmol O_2_/min/mg dry weight), maximal (B) and submaximal (C) mitochondrial H_2_O_2_ emission (pmol H_2_O_2_/min/mg dry weight) together with myofibrillar (D) and sarcoplasmic (E) protein synthesis rates (FSR, %/day) assessed over a 7‐day period using deuterium oxide in C57BL/6NJ (wild‐type, WT, black dots, *n* = 8) and mitochondrial catalase transgenic (MCAT, white dots, *n* = 8) mice. H_2_O_2_ emission and oxidative phosphorylation values were determined under the presence of 5 mM pyruvate, 1 mM malate, and 20 mM succinate, and the absence (maximal H_2_O_2_) or presence of ADP (100 uM for submaximal H_2_O_2_ and oxidative phosphorylation, 10,000 μM for maximal oxidative phosphorylation). Values represent means + SDs. Data were analysed using an unpaired Student's *t* test. *Significantly different from WT (*P* < 0.05).

**Figure 7:**
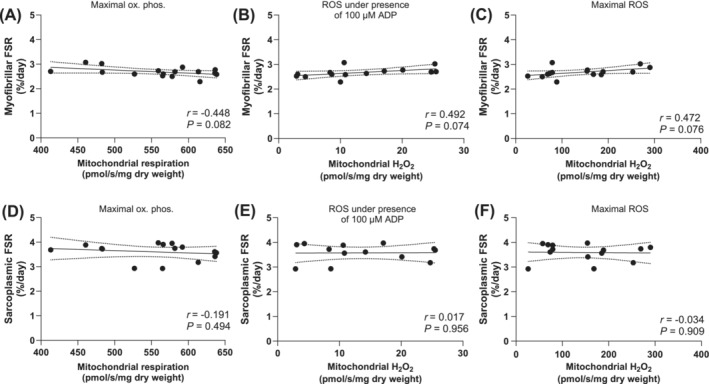
Associations between resting maximal oxidative phosphorylation (A, pmol O_2_/min/mg dry weight), submaximal (B), and maximal (C) H_2_O_2_ emission (pmol H_2_O_2_/min/mg dry weight) and daily myofibrillar protein synthesis rates (FSR, %/day) assessed over a 7‐day period using deuterium oxide in C57BL/6NJ and mitochondrial catalase transgenic mice (*n* = 16). Associations between resting maximal oxidative phosphorylation (D, pmol O_2_/min/mg dry weight), submaximal (E), and maximal (F) H_2_O_2_ emission (pmol H_2_O_2_/min/mg dry weight) and daily sarcoplasmic protein synthesis rates (FSR, %/day). Data were analysed using Pearson's *r* product moment correlations. The solid line represents the linear regression line of best fit and the dashed lines represent the 95% confidence interval.

Given the historical emphasis of mitochondrial redox stress and muscle mass dysregulation, we lastly utilized a transgenic mouse model to solidify the absence of a relationship between mitochondrial H_2_O_2_ and muscle protein synthesis. To achieve this, we investigated mitochondrial bioenergetics and sarcoplasmic and myofibrillar protein fractional synthesis rates in the skeletal muscle of mice overexpressing catalase targeted to the mitochondria (MCAT), which resulted in a phenotype with marked reductions in mitochondrial H_2_O_2_ emission. MCAT mice did not display differences in body mass (WT: 31 ± 3 vs. MCAT: 33 ± 2 g, *P* = 0.199) or oxidative phosphorylation (Figure 6A, *P* = 0.932) compared with WT mice. As expected, maximal (Figure 6B, *P* < 0.0001) and submaximal (Figure 6C, *P* = 0.004) H_2_O_2_ emission were substantially lower in MCAT versus wild type mice. Despite the ~3‐fold lower rates of H_2_O_2_ emission, MCAT mice did not display differences in average daily myofibrillar (Figure 6D, *P* = 0.205) or sarcoplasmic (Figure 6E, *P* = 0.438) protein synthesis rates. Additionally, no associations were detected between maximal oxidative phosphorylation, submaximal or maximal H_2_O_2_ emission and average daily myofibrillar (Figure 7A–C) or sarcoplasmic (Figure 7D–F) protein synthesis rates assessed over a 7‐day period in MCAT and WT mice (all *P* > 0.05).

## Discussion

The present data reveal that intrinsic mitochondrial oxidative phosphorylation and ROS emission are not associated with muscle protein synthesis rates or changes in skeletal muscle mass. Specifically, we show that the intrinsic mitochondrial oxidative phosphorylation and ROS emission are not associated with (1) myofibrillar protein synthesis rates in younger men at baseline, (2) daily skeletal muscle protein synthesis rates or changes in skeletal muscle mass during 1 week of strict bed rest in young men, or (3) skeletal muscle protein synthesis rates or changes in skeletal muscle mass during 12 weeks of resistance‐type exercise training in older men. Supporting these data, we observed (4) that mice possessing dramatically different mitochondrial ROS emission rates do not display differences in myofibrillar and sarcoplasmic protein synthesis rates.

Various human and rodent studies have suggested a key role for mitochondrial bioenergetics in regulating skeletal muscle protein synthesis rates.^34^, ^35^ Here, we applied deuterium oxide over 7 days to integrate the impact of fasting, feeding, physical (in)activity, sleep, and other modulatory factors into a single measurement of muscle protein synthesis rates along with several readouts of basal mitochondrial bioenergetics and markers of muscle redox status (i.e., GSH:GSSG). In contrast to our hypothesis, nearly all readouts of mitochondrial bioenergetics (mass and intrinsic specific) were not associated with muscle protein synthesis rates or skeletal muscle mass across the age spectrum. In older men, only submaximal mitochondrial oxidative phosphorylation (corrected for mitochondrial content) was positively associated with myofibrillar protein synthesis rates during exercise training. Despite this finding, however, we did not observe correlations between changes in any mitochondrial bioenergetic readouts and changes in muscle mass following exercise training in older men. The absence of other correlations across both age categories may be, at least partly, attributed to the inclusion of older individuals who had also completed a 12‐week exercise intervention and, therefore, represent relatively healthy older adults. While these individuals may not fully represent the general older population, this approach was chosen to minimize possible confounding effects related to age‐associated sedentary lifestyle to examine direct effects of chronological aging. Nevertheless, these older individuals displayed reductions in mitochondrial ADP sensitivity,^12^ suggesting that age‐associated changes in mitochondrial bioenergetics are unlikely to impact muscle protein synthesis rates. The absence of associations do not appear to be specific to aging, as physical inactivity induced by short term bedrest decreased markers of mitochondrial content but not myofibrillar protein synthesis,^15^ while mitochondrial oxidative phosphorylation (mass and mitochondrial specific) did not correlate with myofibrillar protein synthesis rates. The lack of clear changes in intrinsic mitochondrial respiratory function with aging and physical inactivity importantly suggests the induction of gene transcription with increased physical activity may manifest only as an improved muscle phenotype. Indeed, several studies have established that increased physical activity enhances mitochondrial content and respiratory function in older individuals.^7^, ^12^, ^36^, ^37^ Furthermore, age‐related declines in mitochondrial content and function are more apparent in studies that did not match for physical activity between young and older individuals.^36^, ^37^, ^38^, ^39^, ^40^ As such, the present findings provide further support that any relationship between mitochondrial oxidative phosphorylation and muscle mass with aging *per se* may be secondary to changes in the level of physical activity that occur over the life span as opposed to a primary cause of age‐induced sarcopenia. We can only speculate whether more severe impairments in mitochondrial respiratory function, along with the consequential reduction in ATP production, have some impact on the regulation myofibrillar protein synthesis rates.

We have recently shown that older individuals display marked ADP insensitivity, manifesting in greater rates of mitochondrial H_2_O_2_ emission.^12^ Given the wealth of data implicating ROS as a key regulator of muscle mass/sarcopenia, we hypothesized that mitochondrial H_2_O_2_ (mass‐specific and/or intrinsic) would associate with myofibrillar protein synthesis rates in diverse aging and physical (in)activity settings. In contrast to our hypothesis, we observed no relationship between myofibrillar protein synthesis and mitochondrial H_2_O_2_ and cellular redox stress in unchallenged young men, following bedrest, or in older men following prolonged exercise training. These data are in general agreement with one human study demonstrating no relationship between redox status (*i.e*., carbonylation) of myofibrillar, sarcoplasmic, or mitochondrial proteins and skeletal muscle mass in young and older men.^41^ While our human data are supported by the previous finding that MnSOD‐deficient mice display oxidative stress without an exacerbation of age‐related muscle mass loss,^42^ several lines of research in other rodent models conflict with the present finding by demonstrating a strong association between increased ROS and lower skeletal muscle mass.^2^, ^3^, ^20^, ^43^ The discrepancy between findings in rodent models and the present study may be explained by more severe dysregulation caused by the far greater capacity for mitochondrial ROS emission and 8‐fold greater rate of muscle protein synthesis in murine models when compared to humans. However, another key finding in the present study is that mice with dramatically different mitochondrial ROS emission rates do not display different rates of myofibrillar protein synthesis. This finding supports the lack of a direct relationship between mitochondrial ROS and myofibrillar protein synthesis rates and changes in quadriceps CSA observed in human muscle. Regardless of the possibility of species differences, the present data suggest that basal mitochondrial redox biology is not a key determinant in the regulation of daily myofibrillar protein synthesis rates and the maintenance of skeletal muscle mass. While mitochondrial ROS likely fluctuates throughout the day in response to macronutrient availability, hormones, and physical activity, these natural oscillations cannot be replicated *in vitro* at a single timepoint. While it is currently impossible to assess mitochondrial ROS emission continuously throughout the day in humans, we have previously shown that bedrest does not alter GSH:GSSG, 4HNE, and protein carbonylation,^15^ markers of *in vitro* redox stress which should reflect daily oscillation in redox stress. Nevertheless, the impact of mitochondrial ROS emission on muscle protein synthesis rates in response to physical activity, food ingestion, and hormones throughout the day, and across the lifespan requires further evaluation.

In conclusion, the present study demonstrates that muscle mitochondrial oxidative phosphorylation and ROS emission do not correlate with skeletal muscle adaptive responses to physical (in)activity in young and older individuals. Changes in mitochondrial oxidative phosphorylation and ROS emission do not correlate with changes in muscle mass that occur in response to short‐term bed rest or resistance‐type exercise training. The lack of any regulatory impact of ROS emission on muscle mass regulation was reinforced by the absence of differences in myofibrillar and sarcoplasmic protein synthesis rates in mice expressing dramatically different mitochondrial ROS emission rates. Overall, the present *in vivo* human and rodent data indicate that mitochondrial oxidative phosphorylation and ROS emission do not appear to play a key role in skeletal muscle mass regulation and the adaptive responses to inactivity and resistance exercise training in younger and older individuals.

## Conflict of interest

The authors declare no competing interests.

## Supporting information


**Table S1.** Subjects' characteristics^1^.

## Data Availability

The data that support the findings of this study are available on request from the corresponding author. The data are not publicly available due to privacy or ethical restrictions.
