# Supplementary material for: Mitochondrial bioenergetics are not associated with myofibrillar protein synthesis rates
Source: J Cachexia Sarcopenia Muscle. 2024 Jul 15;15(5):1811–22. doi: 10.1002/jcsm.13532 (PMC11446679; doi:10.1002/jcsm.13532)
Supplement: Supplementary file 1 — Table S1. Subjects' characteristics1. [file JCSM-15-1811-s001.docx]

| **Change in mixed muscle fiber CSA** |  |  |
| --- | --- | --- |
|  | **P-value** | **r-value** |
| Change in Maximal Oxidative phosphorylation | 0.99 | 0.00 |
| Change in Maximal Oxidative phosphorylation (mtDNA corrected) | 0.42 | -0.29 |
| Change in Sub-maximal Oxidative phosphorylation (25 µM) | 0.07 | -0.60 |
| Change in Sub-maximal Oxidative phosphorylation (25 µM, mtDNA corrected) | 0.14 | -0.50 |
| Change in Maximal H_2_O_2_ | 0.10 | -0.54 |
| Change in Maximal H_2_O_2_ (mtDNA corrected) | 0.61 | -0.19 |
| Change in Sub-maximal H_2_O_2_ (25 µM) | 0.25 | -0.40 |
| Change in Sub-maximal H_2_O_2_ (25 µM, mtDNA corrected) | 0.52 | -0.23 |
| Change in Glutathione | 0.99 | -0.01 |
| Change in GSH | 0.73 | -0.13 |
| Change in GSSG | 0.93 | -0.03 |
| Change in GSH:GSSG | 0.98 | 0.01 |
|  |  |  |
| **Change in Type I fiber CSA** |  |  |
|  | **P-value** | **r-value** |
| Change in Maximal Oxidative phosphorylation | 0.77 | -0.11 |
| Change in Maximal Oxidative phosphorylation (mtDNA corrected) | 0.33 | -0.34 |
| Change in Sub-maximal Oxidative phosphorylation (25 µM) | 0.02 | -0.71 |
| Change in Sub-maximal Oxidative phosphorylation (25 µM, mtDNA corrected) | 0.13 | -0.51 |
| Change in Maximal H_2_O_2_ | 0.08 | -0.58 |
| Change in Maximal H_2_O_2_ (mtDNA corrected) | 0.36 | -0.32 |
| Change in Sub-maximal H_2_O_2_ (25 uM) | 0.19 | -0.45 |
| Change in Sub-maximal H_2_O_2_ (25 uM, mtDNA corrected) | 0.41 | -0.29 |
| Change in Glutathione | 0.96 | -0.02 |
| Change in GSH | 0.94 | 0.03 |
| Change in GSSG | 0.82 | 0.08 |
| Change in GSH:GSSG | 0.71 | -0.13 |
|  |  |  |
| **Change in Type II fiber CSA** |  |  |
|  | **P-value** | **r-value** |
| Change in Maximal Oxidative phosphorylation | 0.82 | -0.08 |
| Change in Maximal Oxidative phosphorylation (mtDNA corrected) | 0.46 | -0.26 |
| Change in Sub-maximal Oxidative phosphorylation (25 µM) | 0.07 | -0.60 |
| Change in Sub-maximal Oxidative phosphorylation (25 µM, mtDNA corrected) | 0.22 | -0.43 |
| Change in Maximal H_2_O_2_ | 0.16 | -0.49 |
| Change in Maximal H_2_O_2_ (mtDNA corrected) | 0.84 | -0.07 |
| Change in Sub-maximal H_2_O_2_ (25 µM) | 0.27 | -0.39 |
| Change in Sub-maximal H_2_O_2_ (25 µM, mtDNA corrected) | 0.73 | -0.13 |
| Change in Glutathione | 0.94 | -0.03 |
| Change in GSH | 0.82 | 0.08 |
| Change in GSSG | 0.74 | -0.12 |
| Change in GSH:GSSG | 0.68 | 0.15 |

**Supplemental Table 1**. Subjects’ characteristics^1^

^1^Values are derived from Pearson’s *r* moment correlations conducted on data of the older individuals (*n* = 10). H_2_O_2_ emission and oxidative phosphorylation values were determined under the presence of 5 mM Pyruvate, 1 mM Malate, and 20 mM Succinate, and the absence (maximal H_2_O_2_) or presence of ADP (25 µM for sub-maximal H_2_O_2_ and oxidative phosphorylation, 10000 µM for maximal oxidative phosphorylation). CSA, cross-sectional area; mtDNA, mitochondrial DNA. Significance was set at *P* < 0.05.
